# Oxidative Biomarkers Associated with the Pulmonary Manifestation of Post-COVID-19 Complications

**DOI:** 10.3390/jcm12134253

**Published:** 2023-06-25

**Authors:** Kamil Siekacz, Anna Kumor-Kisielewska, Joanna Miłkowska-Dymanowska, Małgorzata Pietrusińska, Krystian Bartczak, Sebastian Majewski, Adam Stańczyk, Wojciech J. Piotrowski, Adam J. Białas

**Affiliations:** 1 Department of Pneumology, Medical University of Lodz, 90-419 Lodz, Poland; kamil.siekacz@stud.umed.lodz.pl (K.S.);; 2 Department of Clinical Pharmacology, Medical University of Lodz, 90-419 Lodz, Poland; 3 Department of Pulmonary Rehabilitation, Regional Medical Center for Lung Diseases and Rehabilitation, Blessed Rafal Chylinski Memorial Hospital for Lung Diseases, 91-520 Lodz, Poland

**Keywords:** SARS-CoV-2, post-COVID-19, PINK1, DNM1L, CCL18, TNF-α, IFN-α, sRAGE, pulmonary fibrosis, mitophagy, oxidative stress

## Abstract

Introduction: The role of mitochondria in post coronavirus disease 2019 (post-COVID-19) complications is unclear, especially in the long-term pulmonary complications. This study aims to investigate the association between post-COVID-19 pulmonary complications and mitochondrial regulatory proteins in the context of oxidative stress. Methodology: Patients who had recovered from COVID-19 were enrolled. According to the evidence of persistent interstitial lung lesions on computed tomography (CT), patients were divided into a long-term pulmonary complications group (P(+)) and a control group without long-term pulmonary complications (P(−)). We randomly selected 80 patients for investigation (40 subjects for each group). Biomarkers levels were determined by enzyme-linked immunosorbent assay (ELISA). Results: The serum concentrations of mitochondrial regulatory proteins were significantly higher in the P(+) group, including PTEN-induced kinase 1 (PINK1): 1.62 [1.02–2.29] ng/mL vs. 1.34 [0.94–1.74] ng/mL (*p* = 0.046); Dynamin-1-like protein (DNM1L): 1.6 [0.9–2.4] ng/mL IQR vs. 0.9 [0.5–1.6] ng/mL (*p* = 0.004); and Mitofusin-2 (MFN2): 0.3 [0.2–0.5] ng/mL vs. 0.2 [0.1–0.3] ng/mL IQR (*p* = 0.001). Patients from the P(+) group also had higher serum levels of chemokine ligand 18 (PARC, CCL18), IL-6, and tumour necrosis factor-alpha (TNF-α) cytokines than the P(−) group. The concentration of interferon alpha (IFN-α) was decreased in the P(+) group. Furthermore, we observed statistically significant correlations between the advanced glycation end product (sRAGE) and TNF-α (Pearson’s factor R = 0.637; *p* < 0.001) and between serum levels of DNM1L and IFN-α (Pearson’s factor R = 0.501; *p* = 0.002) in P(+) patients. Conclusions: Elevated concentrations of mitochondrial biomarkers in post-COVID-19 patients with long-term pulmonary complications indicate their possible role in the pathobiology of COVID-19 pulmonary sequelae. Oxidative stress is associated with the immune response and inflammation after COVID-19. TNF-α could be a promising biomarker for predicting pulmonary complications and may be a potential target for therapeutic intervention in patients with post-COVID-19 complications.

## 1. Introduction

COVID-19 is a severe respiratory disease that has a wide spectrum of manifestations, from asymptomatic forms to acute respiratory failure with multiple organ malfunctions [1,2]. The etiological agent of the disease is a novel positive single-strand RNA virus known as severe acute respiratory syndrome coronavirus 2 (SARS-CoV-2) (4).

In cells, SARS-CoV-2 replicates causing tissue damage. The prognosis of COVID-19 depends on a wide range of factors including age, comorbidities, health condition, time from disease onset to treatment, and response to therapy [3,4]. The disease has a variety of long-term complications comprising pulmonary complications, especially lung fibrosis [5,6,7].

Post-COVID-19 lung fibrosis refers to the scarring or fibrosis of lung tissue that occurs after recovery from COVID-19 [8]. This condition is caused by the immune response to SARS-CoV-2, which can lead to inflammation and damage in the lungs. Activated T lymphocytes, monocytes, and neutrophils release cytokines including TNF-α, CCL18 (PARC), granulocyte-macrophage colony-stimulating factor (GM-CSF), IL-1, IL-6, and interferons [9,10,11]. Prolonged inflammation and immunological imbalance can result in the formation of scar tissue, which can impair the ability of the lungs to function properly, leading to shortness of breath, coughing, and fatigue. Post-COVID-19 lung fibrosis may take several months or longer to develop after the initial infection [12]. According to the European Respiratory Society Statement, post-COVID-19 complications are defined as “signs and symptoms that continue or develop after acute COVID-19 and post-COVID-19 syndrome, encapsulates those with symptoms persisting >12 weeks” [13].

SARS-CoV-2 replication products were found in mitochondria and are in involved in mitochondrial homeostasis [14]. Moreover, mitochondrial dysfunction is associated with post-COVID-19 complications. Impaired mitochondrial bioenergetic factors including anaerobic glycolysis, lactate, and reactive oxygen species (ROS) formation are possible explanations for chronic inflammation and post-COVID-19 disease [15]. Dysfunctional mitochondria should be removed in a process called mitophagy. Mitophagy is a type of autophagy that plays a crucial role in maintaining mitochondrial quality control, eliminating dysfunctional mitochondria and promoting cellular homeostasis. In COVID-19, this process is reduced [14]. Mitophagy depends on the expression of several proteins including PINK1, DNM1L, and mitofusin-1 and 2 (MFN1 and 2) [16]. 

SARS-CoV-2 infection causes oxidative stress. Elevated ROS leads to post-COVID-19 complications [17]. Both plasma endothelial and oxidative stress biomarkers were associated with late mortality in hospitalized COVID-19 patients [18]. Induced inflammation reinforced by oxidative stress contributes to post-COVID-19 complications [19]. Moreover, antioxidant markers are overexpressed in COVID-19 patients [20]. Therefore, antioxidants might reduce the development of post-COVID-19 complications [18].

Interestingly, a high level of soluble receptors for sRAGE is correlated with the risk of COVID-19 mortality [21]. RAGE is mainly expressed in the lungs, especially in oxidative stress situations. sRAGE could be involved in pulmonary fibrosis [22]. sRAGE recognizes multiple ligands, for example, advanced glycation end products (AGEs), S100 proteins, amyloid β and amyloid fibrils, high mobility group box-1 protein (HMGB1), and β2-integrins. RAGE–ligand binding elicits an immune reaction [23,24].

This study aimed to investigate the association between mitochondrial regulatory proteins and post-COVID-19 pulmonary complications in the context of oxidative stress. Our research focused on the significance of oxidative stress and its involvement in immune response and inflammation after COVID-19.

## 2. Materials and Methods

### 2.1. Subjects

**Scheme 1:**
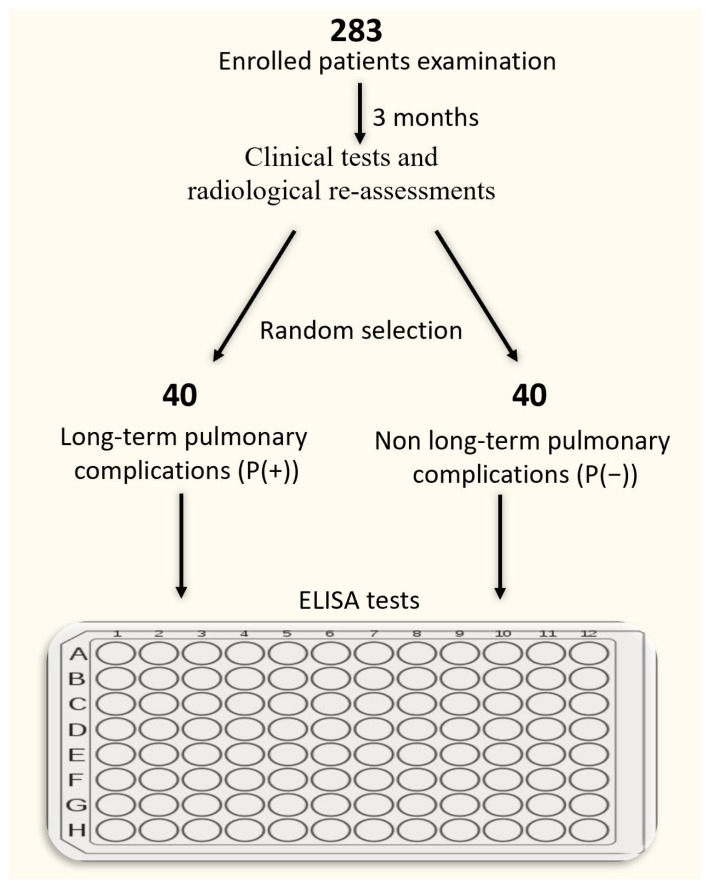
Experiment design and patient selection for the ELISA tests.

We enrolled 283 adult patients from the Outpatient Clinic and Department of Pneumology of the Medical University of Lodz from 2020 to the end of 2021 who had recovered from COVID-19. In all patients, SARS-CoV-2 infection was confirmed by a real-time polymerase chain reaction (RT-PCR) test. There were no specific exclusion criteria. From the entire study cohort, we selected randomly 80 patients for further investigation (40 subjects for each group) as described below. The pulmonary manifestation of post-COVID-19 complications was defined by lung lesions, with or without impairment noted in pulmonary function tests (PFTs), that persisted after 3 months after recovery from active COVID-19. This group was labelled as P(+). The control group consisted of patients who had recovered from COVID-19 and presented neither lung lesions nor impairment in PFTs in a 3-month follow-up. Patients in this group were selected randomly using the web-based tool served by the research randomizer page (https://www.randomizer.org, accessed on 23 March 2022). This group was labeled as P(−). Smokers and ex-smokers were defined according to the recommendations of the Center for Disease Control and Prevention [25]. The comprehensive assessment of the participants was performed. The experimental design is shown in Scheme 1.

### 2.2. Pulmonary Function Tests

Spirometry and the single-breath transfer factor of the lung for carbon monoxide (TLCO) measurements were performed using a Lungtest 1000 system (MES, Cracow, Poland) according to ATS/ERS standards [26]. Forced expiratory volume in 1 s (FEV1), forced vital capacity (FVC), and TLCO corrected for hemoglobin concentration were recorded. (Table 1).

### 2.3. Samples

Venous blood samples were collected by venipuncture into tubes with K2EDTA and tubes for preparing serum with gel (total volume 4.5 + 5 mL). Samples for serum were allowed to clot and after 30 min were centrifuged at approximately 1000× *g* at 4 °C for 10 min (centrifuge MPW 223e). The serum was then transferred to an Eppendorf tube and stored at −80 °C for later use. Morphology and biochemistry measurements were performed using a Sysmex 2000XN and a Beckman Coulter au480, respectively.

### 2.4. ELISA

Serum levels of PINK1, DNM1L, and MFN1 and 2 were measured using an enzyme-linked immunosorbent assay kit (Cloud-Clone Corp; Houston, TX, USA). To assess the levels of IL-6, IFN-α, and TNF-α cytokines, enzyme-linked immunosorbent assay kits (Diaclone Immunology Product & Services; Besançon; France) were used. sRAGE was also measured using an enzyme-linked immunosorbent assay kit (BioVendor R&D; Brno; Czech Republic). The manufacturer’s protocols were followed. Detection was performed on the microplate reader (Microplate Reader BioTek 800 Elx) and conducted at 450 nm immediately.

### 2.5. Statistical Analysis

Statistical analysis was performed using Statistica v13.3 2017 TIBCO software for Windows OS. Continuous data are presented as the mean with standard deviation (SD) or median with interquartile range (IQR) depending on the distribution of data. Variables were compared using the unpaired Student’s *t*-test, Welch’s *t*-test, or the Wilcoxon rank sum test with continuity correction depending on data normality and homogeneity of variance. Pearson’s correlation was used for correlation analyses. For continuous data analysis, data values under the limit of detection (the LOD) were replaced with the LOD divided by the square root of 2 [27]. Receiver operating characteristic (ROC) analyses were performed in the comparison of the diagnostic value of biomarkers in the prediction of the development of long-term pulmonary lesions in patients who had recovered from COVID-19.

## 3. Results

### 3.1. Characteristics of the Investigated Population

Based on the investigated population, this study revealed differences between groups of individuals. Within the P(+) group, 39 participants required hospitalization, with a median duration of 12.5 days (IQR 7–26 days). Conversely, in the P(−) group, only 5 patients were admitted to the hospital, whereas the remaining 35 individuals received ambulatory care at the outpatient clinic. The vast majority of hospitalized patients underwent treatment involving passive oxygen therapy and the administration of steroids and antibiotics according to their clinical and laboratory findings. Patients from ambulatory care at the outpatient clinic received only symptomatic treatment. Importantly, none of the participants required intensive care unit (ICU) admission. Table 2 characterizes the participants included in the study. Except for sex and active smoking, no statistically significant difference was observed between the investigated groups. The number of male participants was higher in both the P(+) and P(−) groups. Surprisingly, the P(−) group had a significantly higher occurrence of smokers.

### 3.2. ELISA Test Results

**Figure 1:**
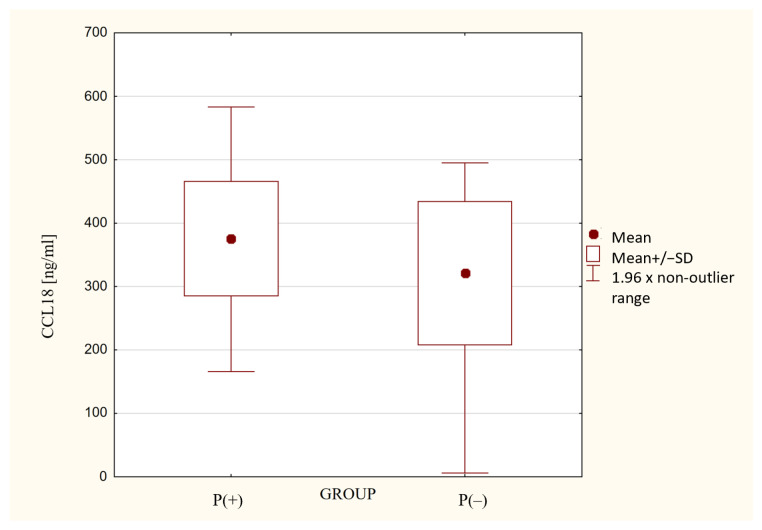
The difference in pulmonary and activation-regulated chemokine (PARC, CCL18) cytokine levels between the investigated groups. P(+)—long-term pulmonary complications group; P(−)—no long-term pulmonary complications group; *p* = 0.036.

Analysis of the ELISA results showed significant differences in serum biomarker concentrations in the investigated groups. Table 3 compares the level of biomarkers in the P(+) and P(−) groups. Values for the MFN1 protein were under the limit of detection in both groups.). Both mitochondrial markers and cytokine levels were higher in the P(+) group. The exception was IFN-α. The median of IFN-α was 4.4 [2.3–7.6] pg/mL in the P(−) group and 2.9 [0.2–5.5] pg/mL (*p* = 0.039) in the P(+) group. Interestingly, the CCL18 (PARC) cytokine results had a normal distribution in the investigated population and there was a significant difference between the P(+) and (P(−) groups. (Figure 1).

**Figure 2:**
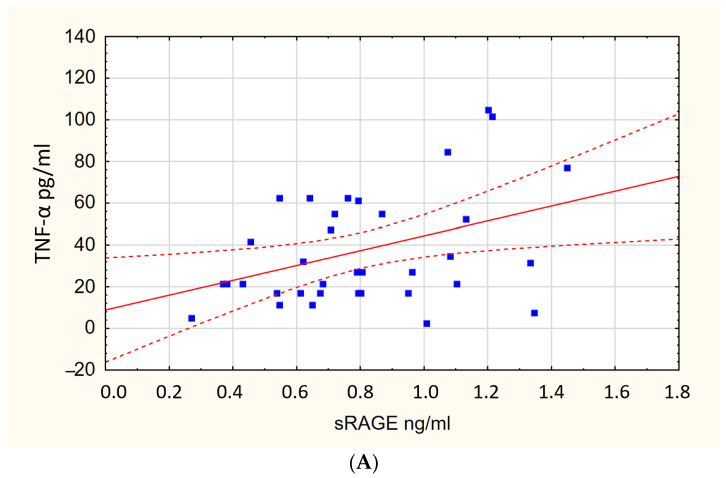

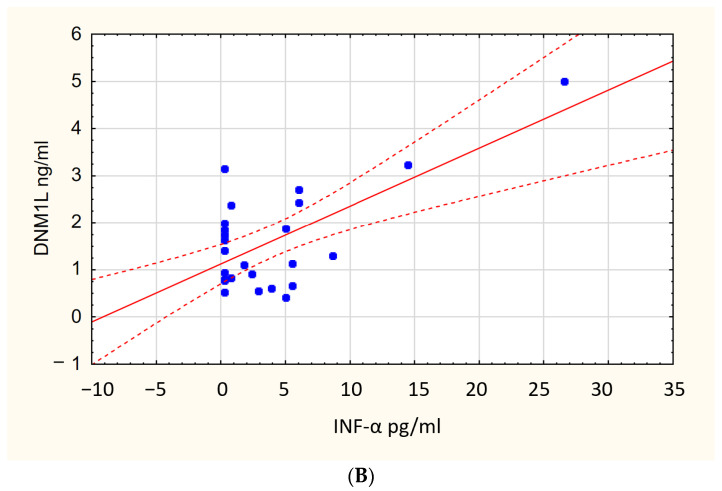
(**A**) Correlation between serum levels of soluble receptor for the advanced glycation end product (sRAGE) and tumor necrosis factor-alpha (TNF-α) in patients with long-term pulmonary complications (P(+)). Pearson’s factor R = 0.637; *p* < 0.001. (**B**) Correlation between serum levels of dynamin-1-like protein (DNM1L) and interferon-alpha (IFN-α) in patients with long-term pulmonary complications (P(+)). Pearson’s factor R = 0.501; *p* = 0.002; red solid line—regression line; red dashed lines—0.95 confidence interval.

**Figure 3:**
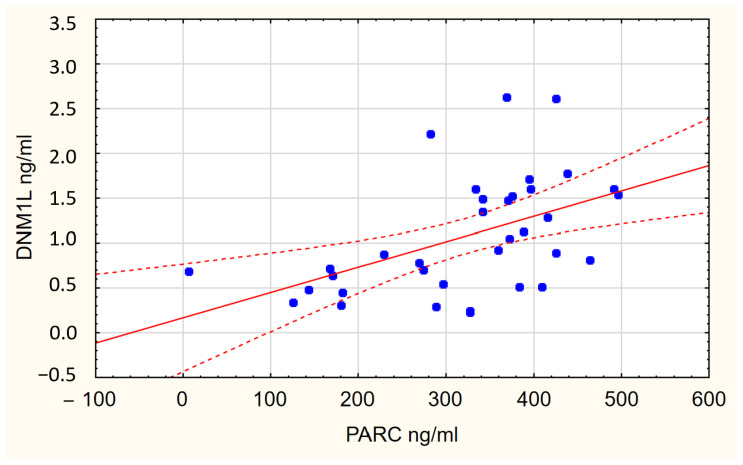
Correlation between serum levels of dynamin-1-like protein (DNM1L) and pulmonary and activation-regulated chemokine (PARC, CCL18) in patients with no long-term pulmonary complications (P(−)). Pearson’s factor R = 0.494; *p* = 0.003; red solid line—regression line; red dashed lines—0.95 confidence interval.

Moreover, we observed a statistically significant correlation between TNF-α and sRAGE serum levels in the P(+) group. The Pearson’s correlation factor was R = 0.637; *p* < 0.001 (Figure 2A). We found a correlation in the P(+) group between serum levels of DNM1L and IFN-α (R = 0.501 *p* = 0.002) (Figure 2B). Moreover, we noted a correlation between serum levels of CCL18 (PARC) and DNM1L in the P(−) group (R = 0.494; *p* = 0.003) (Figure 3).

**Figure 4:**
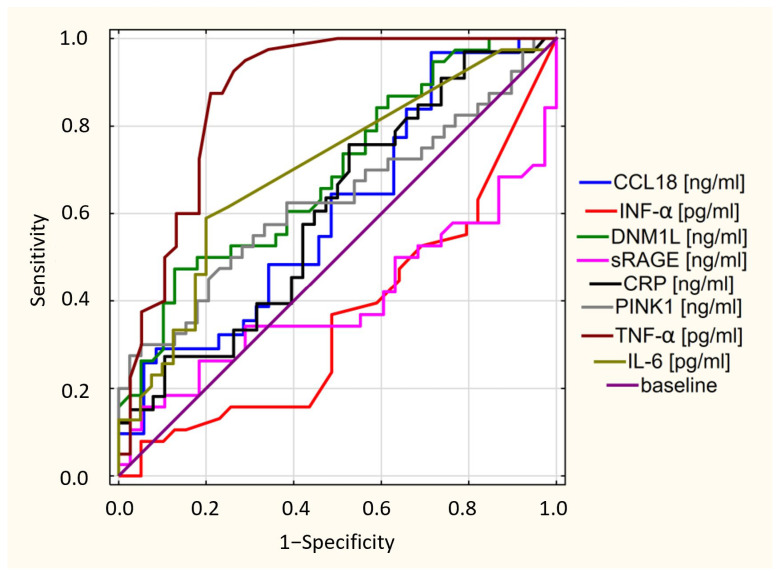
Comparison of the diagnostic value of biomarkers in prediction of the development of long-term pulmonary complications in patients who had recovered from COVID-19.

According to biomarker concentrations in the patients who had recovered from COVID-19, we performed receiver operating characteristic analysis (ROC curves). Only TNF-α had a Youden’s index (Ji = sensitivity + specificity − 1) above 0.5. The other biomarkers had no diagnostic value in the prediction of long-term pulmonary complication development (Figure 4).

## 4. Discussion

The prognosis of COVID-19 depends on a wide range of factors including comorbidities, general health conditions, age, hospitalization time, and ICU admission [28]. Our results support the hypothesis that sex could also be considered a significant factor in the development of post-COVID-19 pulmonary fibrosis. Several studies have shown that males have an increased likelihood of developing pulmonary fibrosis following COVID-19 infection compared with females [29,30]. Contrary to prior research, our results suggest active smoking might be a protective factor for post-COVID-19 pulmonary fibrosis. The literature is inconsistent regarding the impact of gender and smoking status on the development of post-COVID-19 pulmonary fibrosis. The meta-analysis of several risk factors including male gender and smoking status suggests that these factors may not play a significant role in predisposing individuals to the development of pulmonary fibrosis following COVID-19 infection [12].

Understanding the specific mechanisms underlying mitochondrial involvement in post-COVID-19 complications is still an area of research. In the context of COVID-19, several studies have suggested that the virus can impact mitochondrial function and dynamics. During the acute phase of SARS-CoV-2 infection, viral proteins localised in mitochondria impair cellular metabolism. That triggers several effects in mitochondria: (1) increased ROS levels, (2) perturbation of Ca^2+^ signalling, (3) changes in mitochondrial DNA (mtDNA) copy number, (4) decreased mitochondrial membrane potential (MMP) and mitochondrial mass, and (5) induction of mitophagy [31,32,33]. These factors can impact the kinetics of mitochondrial fusion and fission, as well as the size, structure, and distribution of mitochondria within the host cells that are infected by the virus [34]. Therefore, the role of mitochondria and oxidative stress in post-COVID-19 pulmonary complications is compound. We attempt to indicate mitochondrial biomarkers involved in post-COVID-19 pulmonary fibrosis development.

Previous studies have demonstrated that COVID-19 causes both mitochondrial impairment and oxidative stress [35,36]. SARS-CoV-2 replication products are present in mitochondria. The virus “manipulates” the metabolism of the host via ACE2 receptors and open reading frame (ORF) regulation [34] Additionally, mitophagy impairment is associated with SARS-CoV-2 infection [14]. Coronaviruses were found to trigger mitophagy as a strategy to escape host immune responses and facilitate the completion of the viral life cycle. Moreover, a study revealed that viral proteins counteracted the stress caused by a viral infection, leading to the fragmentation and aggregation of mitochondria [37]. In people with suboptimal mitochondrial function, there is a predisposition to post-COVID-19 complications. The possible explanation for this issue is that viral infection stress activates a hermetic negative feedback mechanism that causes a chronic inflammatory cycle [15]. In addition, damaged mitochondria release mtDNA. The abovementioned processes promote mitochondria-derived ROS release, leading to the activation of inflammatory pathways and the release of cytokines. [38]. In the P(+) group, we noticed an increased levels of PINK1—the main protein responsible for the detection of damaged mitochondria. PINK1 marks impaired mitochondria and initiates a degradation cascade [39]. Mitophagy requires the separation of impaired mitochondria via fission. Consequently, the levels of the DNMlL protein were also increased in the P(+) group [40]. Thus, altered mitochondria balance is involved in post-COVID-19 complications [41].

Infection with SARS-CoV-2 induces mitochondrial imbalance to stimulate and sustain chronic inflammation [15,40]. Elevated levels of cytokines and ROS formation in the early stages of COVID-19 influence the development of post-COVID-19 pulmonary complications [42]. These data are consistent with Zong et al., who demonstrated the elevation of TGF-β and CCL18 in patients who had recovered from COVID-19 but had pulmonary fibrosis [4]. Contrary to the abovementioned cytokines, IFN-α was higher in the P(−) group. These results agree with studies on persistent immunological dysfunction in COVID-19 [43]. Interferons are involved in the immunopathogenesis of post-COVID-19 syndrome. Insufficient interferon production may be associated with the development of complications after recovering from COVID-19 [44]. Decreased levels of interferons could be a risk factor for post-COVID-19 pulmonary complications [44,45]. Elevated levels of circulating cytokines, excluding interferons, could induce post-COVID-19 pulmonary fibrosis [46], although the possible role of interferons in antifibrotic treatment is unclear [47]. The ELISA results from our study validate an immune imbalance, as evidenced by elevated cytokine levels in the P(+) group. Notably, serum levels of IFN-α were found to be higher in the P(−) group. Intriguingly, we detected correlations between DNM1L and IFN-α and between TNF-α and sRAGE in the P(+) group.

TNF-α is one of the major cytokines responsible for post-COVID-19 pulmonary fibrosis [7]. We observed that TNF-α could predict long-term pulmonary complications in patients who had recovered from COVID-19. Increased thresholds for TNF-α and IL-6 have already been considered as risk factors for pulmonary fibrosis development [48]. Furthermore, inhibitors of the TNF-α and IL-6 pathways could have therapeutic potential in the treatment of severe COVID-19 and prevent long-term fibrotic consequences [48,49].

Another important finding was the correlation between TNF-α and sRAGE. This observation may support the hypothesis that alveolar epithelial damage could be associated with persistent inflammation [50]. sRAGE has the potential to be of prognostic value for clinical COVID-19 severity [21]. sRAGE could reprogram lung cell metabolism after SARS-CoV-2 infection. Therefore, the sRAGE pathway may be a link between oxidative stress, mitochondria, and long-term pulmonary complications [51,52]. The chronic expression of cytokines is crucial for the maintenance of RAGE activation and the persistence of post-COVID-19 complications [53].

Overall, our study’s strengths lie in its comprehensive analysis of immune and mitochondrial dysregulation in post-COVID-19 pulmonary complications; it also provides potential prognostic biomarkers and suggests possible therapeutic targets for preventing post-COVID-19 pulmonary fibrosis. This research identifies several potential biomarkers that may be useful for predicting and monitoring post-COVID-19 complications. However, it is important to note that further research is needed to fully elucidate the specific roles and mechanisms of mitochondria, mitophagy, and oxidative stress in post-COVID-19 pulmonary complications. The next milestone could be the analysis of microRNA (miRNA) profiles in patients who have recovered from COVID-19. miRNAs play crucial roles in gene expression regulation. Several studies have explored the association between specific miRNAs and factors including prognosis, prediction of disease severity, and complications in patients who have recovered from COVID-19 [54]. Specific miRNAs have been found to correlate with the severity of lung injury and the development of post-COVID-19 pulmonary fibrosis. These miRNAs are associated with fibroblast proliferation and differentiation and the regulation of fibrotic signaling pathways [55]. Additionally, miRNAs are involved in the regulation of mitochondrial function and cellular processes relevant to lung homeostasis. Therefore, dysregulated miRNA profiles were found in the lung tissues of individuals with post-COVID-19 lung injury. Moreover, miRNA dysregulation has been associated with mitochondrial dysfunction, oxidative stress, and inflammation [56,57].

Despite the novel findings of the present study, it has several limitations. It was limited by the absence of a healthy control group and the small size of the investigated groups. Due to technical reasons, some clinical and laboratory data were not available or were limited. The generalizability of these results is subject to specific limitations: the heterogeneity of patients and their treatments, the diversity of pulmonary lesions, and the severity of acute infection. The abovementioned factors might have introduced a selection bias for some data.

## 5. Conclusions

In this study, we have attempted to identify predictors for the development of post-COVID-19 pulmonary complications, especially in the context of oxidative stress. Elevated concentrations of mitochondrial biomarkers in patients who have recovered from COVID-19 but have long-term pulmonary complications indicate their possible role in the pathobiology of COVID-19 pulmonary sequelae. The results from this study suggest that SARS-CoV-2 infection could be involved in mitochondrial imbalance via PINK1, DNM1L, and MFN2 dysregulation. COVID-19 could induce mitochondrial impairment and chronic inflammation with oxidative stress.

Elevated levels of CLL18, TNF-α, and IL-6 could be associated with the development of long-term pulmonary complications after COVID-19. TNF-α might be a potential predictor of pulmonary complications. Additional research is warranted to validate the present study findings.

## Figures and Tables

**Table 1:** Characteristics of pulmonary function in the study groups.

Parameter	P(+) *n* = 36		P(−) *n* = 37		
	Volume (L)	% of Predicted Value *	Volume (L)	% of Predicted Value *	* *p*-Value
FVC, mean (SD)	3.2 (1.24)	74 (20.2)	3.8 (0.99)	92 (11.56)	<0.001
FEV1, mean (SD)	2.6 (0.95)	76 (18.45)	3 (0.72)	90 (12.28)	<0.001
TLCO, mean (SD)	5.7 (2.56)	67 (25.67)	7.9 (2.13)	97 (12.87)	<0.001

Abbreviations: FVC, forced vital capacity; FEV1, forced expiratory volume in one second; TLCO, single-breath transfer factor of the lung for carbon monoxide; P(+), long-term pulmonary complications group; P(−) no long-term pulmonary complications group; * statistically significant difference *p* < 0.05.

**Table 2:** Characteristics of the study groups.

	P(+) *n* = 40	P(−) *n* = 40	*p*-Value
Age (years), mean (SD)	56 (12.21)	53 (11.3)	0.210
Male sex, n (%)	31 (77.5%)	21 (52%)	0.019
BMI (kg/m2), mean (SD)	28 (5.28)	28 (4.7)	0.699
Comorbidities, n (%)			
Hypertension	18 (45%)	14 (35%)	0.367
Obesity	10 (25%)	7 (17.5%)	0.419
Heart failure	5 (12.5%)	1 (2.5%)	0.094
Type 2 diabetes	4 (10%)	8 (20%)	0.216
Asthma	4 (10%)	4 (10%)	0.992
Coronary heart disease	4 (10%)	2 (5%)	0.404
Pulmonary hypertension	2 (5%)	1 (2.5%)	0.569
Smoking			
Active smokers, n (%)	2 (5%)	8 (20%)	0.024
Pack-years, mean (SD)	16 (13)	11 (13)	0.118
Ex-smokers, n (%)	22 (55%)	18 (45%)	0.420

Abbreviations: BMI, body mass index; SD, standard deviation; P(+), long-term pulmonary complications group; P(−), no long-term pulmonary complications group.

**Table 3:** Comparison of biomarker concentrations between investigated groups.

Biomarker	sRAGE [ng/mL]	IL-6 [pg/mL]	IFN-α [pg/mL]	TNF-α [pg/mL]	PINK1 [ng/mL]	DNM1L [ng/mL]	MFN2 [ng/mL]
P(+)	0.77 [0.61–1.07]	1.0 [0.1–75.7]	2.9 [0.2–5.5]	32.4 [2.8–318]	1.62 [1.02–2.29]	1.6 [0.9–2.4]	0.3 [0.2–0.5]
P(−)	0.88 [0.7–1.03]	0.5 [0.14–18.1]	4.4 [2.3–7.6]	2.4 [2.0–158]	1.34 [0.94–1.74]	0.9 [0.5–1.6]	0.2 [0.1–0.3]
*p*-Value	0.135	0.002	0.039	<0.001	0.046	0.004	0.001

Abbreviations: soluble receptor for the advanced glycation end product (sRAGE); interleukin-6 (IL-6); interferon-alpha (IFN-α); tumor necrosis factor-alpha (TNF-α); PTEN-induced kinase 1 (PINK1); dynamin-1-like protein (DNM1L); mitofusin-2 (MFN2); long-term pulmonary complications group (P(+)); no long-term pulmonary complications group (P(−)); in square brackets [IQR]; *p*-Value < 0.05 represents a statistically significant difference.

## Data Availability

Not applicable.
